# Transcriptome and Physio-Biochemical Profiling Reveals Differential Responses of Rice Cultivars at Reproductive-Stage Drought Stress

**DOI:** 10.3390/ijms24021002

**Published:** 2023-01-05

**Authors:** Simardeep Kaur, Karishma Seem, Naveen Duhan, Suresh Kumar, Rakesh Kaundal, Trilochan Mohapatra

**Affiliations:** 1 Division of Biochemistry, ICAR-Indian Agricultural Research Institute, New Delhi 110012, India; 2 Department of Plants, Soils, and Climate, College of Agriculture and Applied Sciences, Utah State University, Logan, UT 84322, USA; 3 Bioinformatics Facility, Center for Integrated BioSystems, College of Agriculture and Applied Sciences, Utah State University, Logan, UT 84322, USA; 4 Indian Council of Agricultural Research, New Delhi 110012, India

**Keywords:** drought stress, transcriptome analysis, rice, reproductive-stage drought, transcription factors, MapMan

## Abstract

Drought stress severely affects the growth and development of rice, especially at the reproductive stage, which results in disturbed metabolic processes, reduced seed-set/grain filling, deteriorated grain quality, declined productivity, and lower yield. Despite the recent advances in understanding the responses of rice to drought stress, there is a need to comprehensively integrate the morpho-physio-biochemical studies with the molecular responses/differential expression of genes and decipher the underlying pathways that regulate the adaptability of rice at various drought-sensitive growth stages. Our comparative analysis of immature panicle from a drought-tolerant (Nagina 22) and a drought-sensitive (IR 64) rice cultivar grown under control (well-watered) and water-deficit/drought stress (treatment, imposed at the reproductive stage) conditions unraveled some novel stress-responsive genes/pathways responsible for reproductive-stage drought stress tolerance. The results revealed a more important role of upregulated (6706) genes in the panicle of N 22 at reproductive-stage drought stress compared to that (5590) in IR 64. Functional enrichment and MapMan analyses revealed that majority of the DEGs were associated with the phytohormone, redox signalling/homeostasis, secondary metabolite, and transcription factor-mediated mitigation of the adverse effects of drought stress in N 22. The upregulated expression of the genes associated with starch/sucrose metabolism, secondary metabolites synthesis, transcription factors, glutathione, linoleic acid, and phenylalanine metabolism in N 22 was significantly more than that in the panicle of IR 64. Compared to IR 64, 2743 genes were upregulated in N 22 under control conditions, which further increased (4666) under drought stress in panicle of the tolerant cultivar. Interestingly, we observed 6706 genes to be upregulated in the panicle of N 22 over IR 64 under drought and 5814 genes get downregulated in the panicle of N 22 over IR 64 under the stress. In addition, RT-qPCR analysis confirmed differential expression patterns of the DEGs. These genes/pathways associated with the reproductive-stage drought tolerance might provide an important source of molecular markers for genetic manipulation of rice for enhanced drought tolerance.

## 1. Introduction

Global climate change has caused erratic weather conditions resulting in a more frequent occurrence of abiotic stresses of varying intensity. Among all the abiotic stresses, drought is one of the serious environmental stresses that affect plant growth, development, and productivity; thus, hsampering crop yield and productivity of several important food crops. This causes ecological imbalance and raises concerns about food/feed security the world over [1,2]. The rice cultivated in rain-fed conditions is severely affected by drought due to reduced precipitation and unpredictable rainfall [3]. The problems are getting aggravated because of global climate change [4,5].

Rice is one of the most important cereal crops cultivated worldwide and constitutes a primary source of human food as it accounts for one-fifth of the total caloric intake by the global human population and provides food for about half of the global population [6,7]. Rice, being a gluten-free, fat-free, cholesterol-free food, and naturally low in sodium content, has become a highly beneficial commodity as a part of a healthy diet for the growing population of people suffering from Celiac disease, Coronary artery disease, Heart disease, Blood pressure, etc. [8]. Due to the rapidly increasing population, rice production would require to be increased by 60% to feed the global population by 2050. Rice, being a water-loving crop, requires plenty of water compared to several other cereal crops [9]. Hence, drought stress, particularly at the reproductive stage (panicle initiation), severely reduces its yield in all agroclimatic regions of the world. Rice cultivation, particularly by transplanting, consumes a major portion of fresh water resulting in limited availability of water for the irrigation of other crops, mainly in the year of low rainfall [10]. Therefore, the need of the day is to develop crop varieties and cultivation practices towards the realization of the proverb “*More Crop per Drop*” to sustain rice cultivation/production [11].

Drought stress induces the production of reactive oxygen species (ROS) and excessive ROS affect signal transduction, stomatal activity, photosynthesis, plant development, and seed set. However, to cope with drought stress, plants have a series of defensive pathways, such as the enzymatic (superoxide dismutase (SOD), catalase (CAT), and ascorbate peroxidase (APX)) and non-enzymatic (carotenoids, flavonoids, ascorbate and glutathione) antioxidants for scavenging of ROS [10,12]. Biosynthesis and accumulation of free proline (an amino acid) in plant tissue during abiotic stress is considered to be an adaptive response [13]. Free proline acts as a signaling moiety against abiotic stresses to stimulate mitochondrial functioning, altering cell proliferation, and activating the stress-responsive genes. It also acts as an excellent osmolyte, metal chelator, and antioxidative defense molecule; thereby, maintaining osmotic balance, membrane integrity and concentrations of ROS within a normal range to prevent oxidative bursts in plants under the stress [14]. Thus, proline content in plant tissue can be used as one of the biochemical markers in assessing the level of abiotic stress the plant might be facing at the moment [15]. A variety of transcription factors (TFs) such as WRKY, ARF, ERF, MYB, etc. play important roles in managing drought stress in plants [1,4,16,17]. Phytohormones, such as ABA, promote root system architecture (RSA) and raise hydraulic conductivity which allows plants to overcome the shortage of water [18]. Stomatal closure affected by ABA under drought stress reduces the loss of water by lowering the rate of transpiration.

Deciphering the molecular basis of abiotic stress tolerance, particularly during the reproductive-stage drought stress, has become easier with the advances in contemporary high-throughput sequencing and bioinformatic analysis software/tools [16]. However, understanding the stress tolerance mechanisms which are active and effective at the most appropriate stage of plant growth requires proper planning/setup of the experiment, selection/collection of the plant tissue(s), comparative analyses of the data for identification of stress-responsive/regulatory genes, pathways, functional characterization, and experimental validation of the genes involved.

Several abiotic stress-associated genes have been reported over the last decade based on comparative expression profiling of stressed versus non-stressed plants/tissues [1,4,10,16,19,20,21]. Although global gene expression analysis in rice under drought stress has been performed in different tissues using the RNA-seq approach [2,4,11,22,23], most of the studies were conducted at early (seedling or vegetative) stages of plant growth/development using different tissues (leaf and root); consequently, only a little information is available about the tolerance mechanisms active/effective against reproductive-stage drought occurring at panicle (inflorescence, an agronomically/economically important tissue that bears spikelets which develop into rice grains) initiation stage (most sensitive/critical stage of drought stress in rice). Still, only little is known about the regulation of reproductive-stage drought stress in rice, particularly about the sensing/signal transduction pathways and their interactions with the genes/pathways that affect seed development and grain yield. Comparative and comprehensive gene expression analysis using drought-tolerant and -sensitive cultivars, grown under simulated/controlled conditions, provides a promising way to decipher the differentially expressed genes (DEGs) and the regulatory/metabolic pathways associated with effective tolerance at reproductive-stage drought stress in rice.

The present study illustrates a comparative and comprehensive genome-wide transcriptome analysis of immature panicles collected from drought-tolerant (Nagina 22, a tall, deep-rooted, drought and heat tolerant *aus* rice cultivar) and drought-sensitive (IR 64, a high-yielding, semi-dwarf, lowland *indica* cultivar with relatively shallow root system) rice cultivars grown under control and water-deficit stress conditions in pots during rice (*Kharif*) season using a rain shelter. Differential expression of genes and their functional enrichment analysis were performed using whole-transcriptome RNA-seq data for the immature panicles collected from the contrasting rice cultivars (an abiotic stresses tolerant N 22 and a rice cultivar highly sensitive to reproductive-stage drought IR 64) subjected to reproductive-stage drought stress as well as grown under control (well-irrigated) conditions. DEG analysis suggests that drought tolerance of N 22 is attributed to the differential expression of genes associated with phytohormone signaling, redox signaling and detoxification, secondary metabolite biosynthesis, and transcription factors. Moreover, MapMan analysis of the transcriptome data (to explore the metabolic pathways and enzyme functions) from two contrasting rice cultivars indicated important roles of phytohormone signalling, redox homeostasis, secondary metabolites, and transcription factor in mitigating the deleterious effects of reproductive-stage drought stress in N 22. The trustworthiness of RNA-seq data and the observed differential expression of genes were validated through RT-qPCR analysis of a few genes. We believe that the selected drought-responsive DEGs associated with defensive metabolic pathways can be exploited for imparting drought tolerance in rice through molecular breeding strategies towards the development of climate-smart cultivars.

## 2. Results

### 2.1. Morpho-Physiological Changes in Contrasting Rice Cultivars under Drought Stress

**Figure 1:**
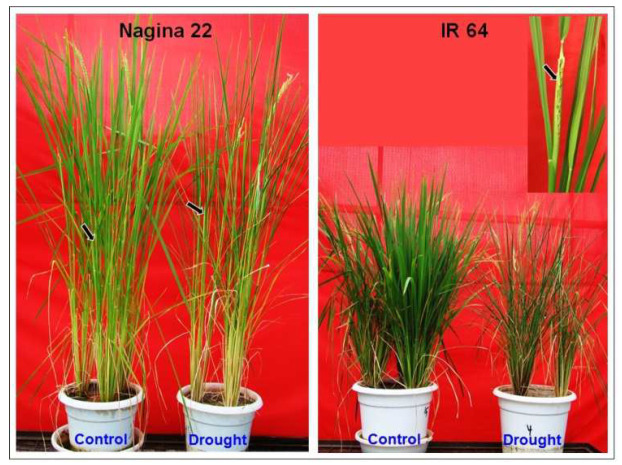
Representative picture of contrasting rice cultivars grown under control and drought stress imposed at the reproductive (panicle initiation) stage. Arrow indicates the panicle (and its stage) collected for biochemical/molecular analysis.

**Figure 2:**
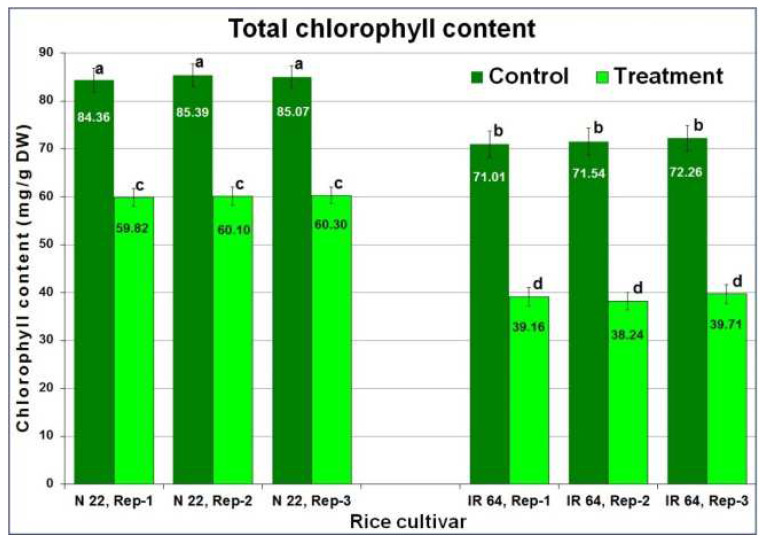
Effect of drought stress on total chlorophyll content in leaf of contrasting rice cultivars. Leaf tissue samples were collected in three biological replications (Rep-1, Rep-2, Rep-3) for the estimation of chlorophyll content. Data present mean value (*n* = 3) for three technical replicates. The mean followed by different lower-case letters is significantly different (*p* ≤ 0.05). The error bar represents the Standard Deviation (±SD).

Imposition of drought stress by withholding irrigation (until soil moisture content (SMC) dropped down to ~6%) resulted in reduced relative water content (RWC) to 58 ± 1% in leaves of IR 64 compared to 61 ± 1% RWC in leaves of N 22. Morphologically, the symptoms of drought stress were observed in the form of rolling and wilting of leaves (Figure 1). Moreover, drought stress caused ~29% reduction in total chlorophyll content in the leaves of N 22 while the reduction was ~45% in the case of IR 64 leaves (Figure 2). Panicle initiation is an important process in the transition from the vegetative stage to the reproductive stage, the effect of reproductive-stage drought stress was assessed on panicle development and seed set/yield. Moreover, the drought stress caused a delay in panicle initiation by 5–7 days.

### 2.2. Biochemical Changes in Contrasting Rice Cultivars on Drought Stress

**Figure 3:**
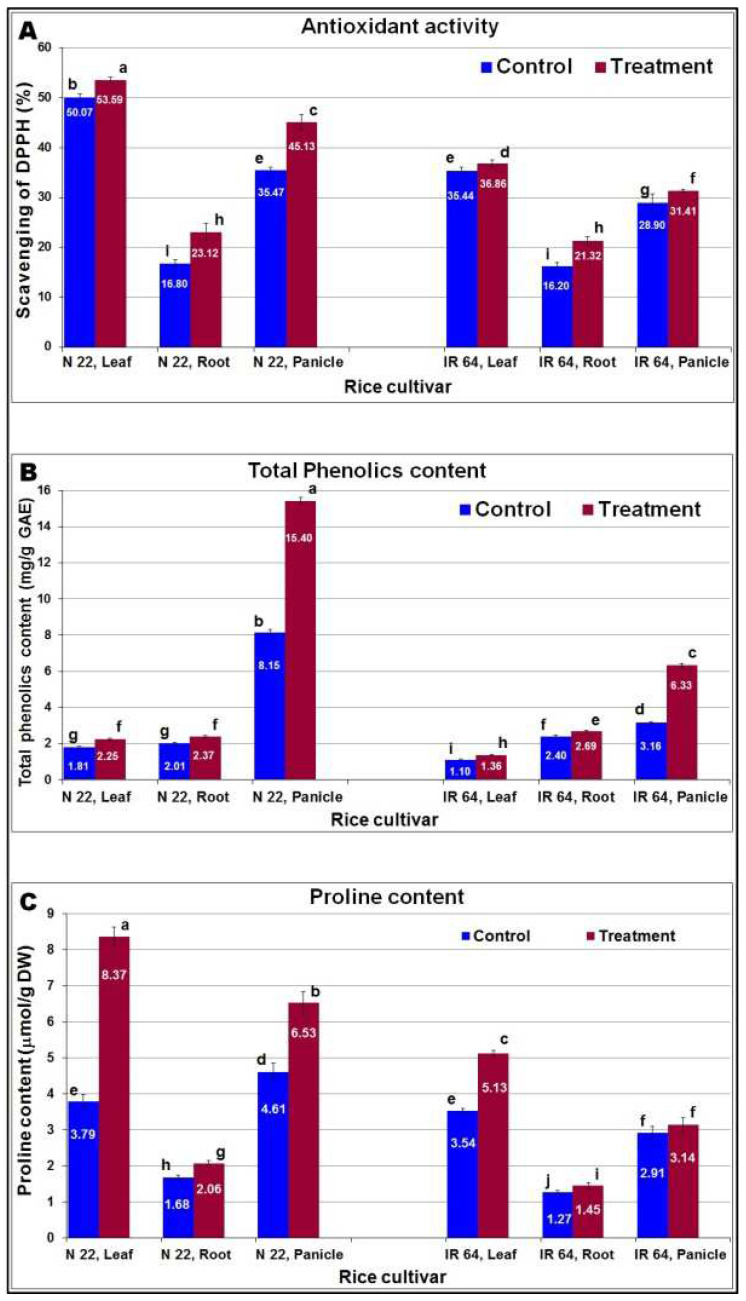
Comparative analysis of changes in biochemical parameters in different tissues (leaf, root, and panicle) of contrasting rice cultivars (N 22 and IR 64) on reproductive-stage drought stress. (**A**) Antioxidant activity (per cent scavenging of DPPH free radical), (**B**) total phenolics content in terms of gallic acid equivalent (GAE), (**C**) proline content in the contrasting rice cultivars. Tissue samples were collected in three biological replications for estimation (in 3 technical triplicates) of the changes in biochemical parameters. Data present mean value (*n* = 9) and the mean followed by different lower-case letters are significantly different (*p* ≤ 0.05). The error bar represents the Standard Deviation (±SD).

Estimation of the effects of reproductive-stage drought on rice plants was assessed based on its impact on biochemical parameters in panicles compared to that in leaf and root. Though antioxidant activity in different tissues of N 22 was higher even under controlled conditions (compared to that in IR 64), drought stress further increased the activity, particularly >27% increase in the immature panicles (Figure 3A). The antioxidant activity is complemented by a considerable (~89%) increase in total phenolics content in the panicle of N 22 compared to that in the panicle of IR 64 (Figure 3B). Protection from the stress was further supplemented by a significant increase in proline content in the leaf and panicle of N 22 compared to that in IR 64 (Figure 3C).

### 2.3. Agronomic Performance of Rice Cultivars under Drought Stress

**Figure 4:**
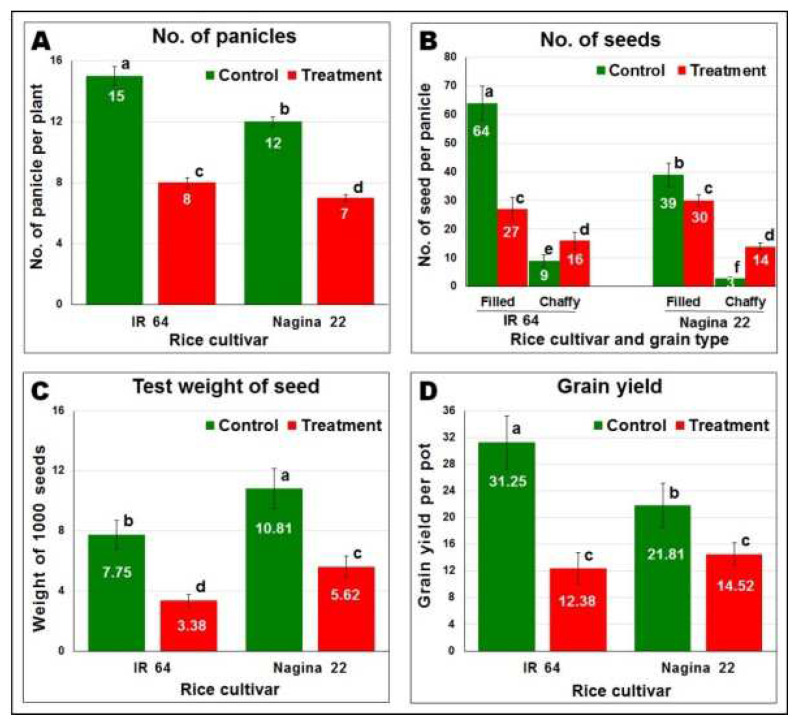
Effect of drought stress on agronomic performance of the rice cultivar. (**A**) Number of panicles per pot, (**B**) grain quality, (**C**) test weight of seed, (**D**) grain yield per pot. Data present mean value (*n* = 3) and the mean followed by different lower-case letters are significantly different (*p* ≤ 0.05). The error bar represents the Standard Deviation (±SD).

A significant decrease in the agronomic performance of the rice cultivar was observed due to reproductive-stage drought stress. More than 46% decrease in the number of panicles in IR 64 compared to that (>41%) in N 22 (Figure 4A) and a significantly increased (>59%) chaffy seeds compared to that (>46) in N 22 (Figure 4B) were recorded under the drought stress. More importantly, the test weight of seeds (the weight of 1000 seeds) was recorded to decrease significantly (Figure 4C). While reduction in the test weight was recorded to be >56% in the case of IR 64, it was ~48% reduction in the case of N 22 under the stress. Accordingly, an overall reduction of >60% in grain yield of IR 64, while ~33% reduction in grain yield of N 22, was recorded due to the reproductive-stage drought stress (Figure 4D).

### 2.4. Library Preparation, Transcriptome Sequencing, and Mapping of Data

**Figure 5:**
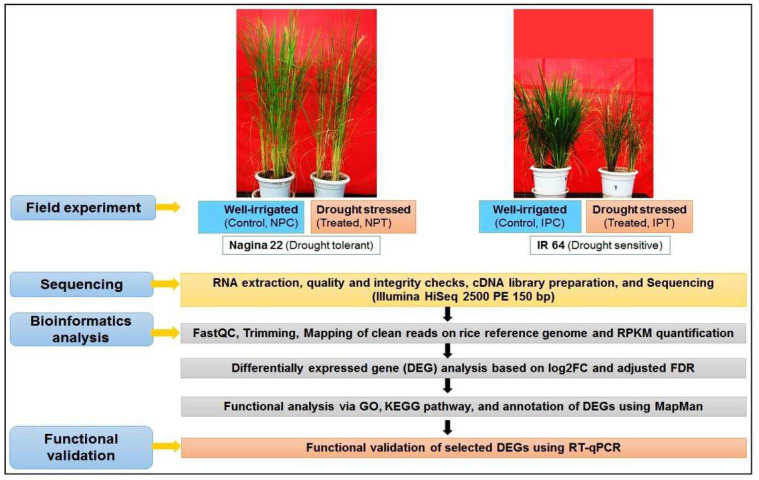
Workflow depicting methodology for the identification of differentially expressed genes (DEGs) and their functional annotation/validation under drought stress in contrasting rice cultivars.

To identify the reproductive-stage drought-responsive genes in the panicle of rice, eight transcriptome libraries were prepared in three replications for panicles from two contrasting rice cultivars grown under control (well-watered) and drought stress [IPC1, IPC2, IPC3 (IR 64 control), and IPT1, IPT2, IPT3 (IR 64, drought treated), NPC1, NPC2, NPC3 (N 22, control), NPT1, NPT2, NPT3 (N 22, drought treated), where ‘P’ denotes panicle tissue]. The libraries were sequenced with the generation of >54 million raw reads for each sample having on an average >92% cleaned reads and >90 mapping efficiency (Table 1). An overview of the steps followed during transcriptome analysis is presented in Figure 5.

### 2.5. Differential Expression of Genes in Contrasting Rice Cultivars 

**Figure 6:**
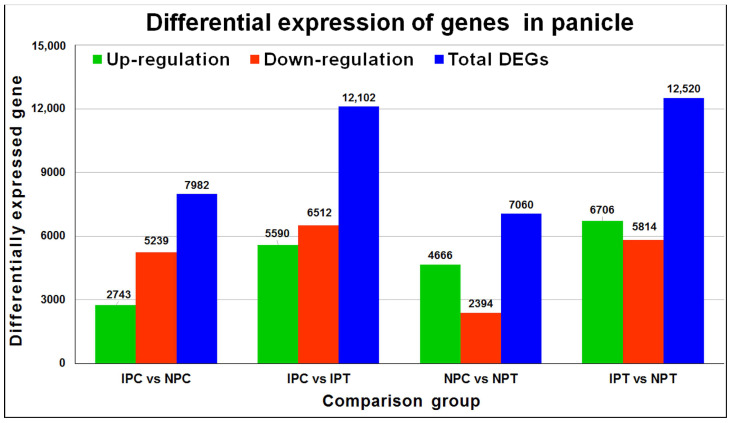
Differentially expressed genes (DEGs) in different comparison groups. IPC: IR 64, panicle, control; IPT: IR 64, panicle, treatment (drought); NPC: Nagina 22, panicle, control; NPT: Nagina 22, panicle, treatment.

To unravel the differential expression of genes in immature panicles of contrasting rice cultivars at reproductive-stage drought stress, the expression level of genes in four comparison groups (IPC vs. IPT, NPC vs. NPT, IPC vs. NPC, and IPT vs. NPT) was analyzed by mapping the reads on rice reference genome available at Ensembl (Figure 6, Supplementary Table S1), wherein the mapping efficiency varied from 83.5 to 93.5% (Table 1) for the clean reads normalized to the reads per kilobase of transcript per million (RPKM) value (Supplementary Table S2).

MA plot analysis [based on Log2 ratio {in minus (M)} on the Y axis and Log2 average (A) on the X axis] for all the significantly up- and downregulated as well as not differentially expressed genes in panicle of the contrasting rice (N 22 and IR 64) cultivars grown under control and drought stress in four different comparison (IPC vs. IPT, NPC vs. NPT, IPC vs. NPC, and IPT vs. NPT) groups (Supplementary Figure S1) indicated that the upregulated genes play important roles in drought tolerance. IR 64 showed more genes to be downregulated in the panicle in response to reproductive-stage drought stress (Supplementary Figure S1A). However, comparatively more genes were upregulated in the panicle of N 22 under the stress (Supplementary Figure S1B). As expected, the number of genes up- and downregulated in the panicle of both the rice cultivars under control conditions (IPC vs. NPC) was almost equal (Supplementary Figure S1C). Moreover, a comparison of the up- and downregulated genes in the panicle of the rice cultivars at reproductive-stage drought stress (IPT vs. NPT) indicated more genes to be upregulated in N 22 (Supplementary Figure S1D).

**Figure 7:**
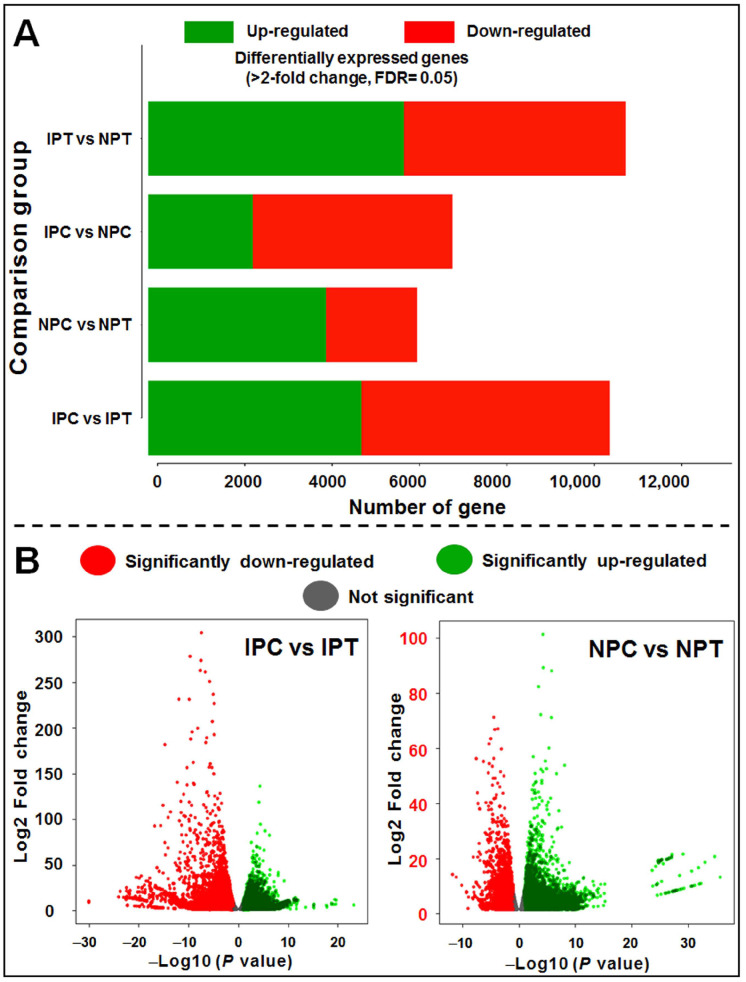
Differentially expressed genes (DEGs) in pairwise comparison with >2-fold change and FDR ≤ 0.05. (**A**) Bar graph depicting significantly up- and downregulated genes in four different comparison groups, (**B**) volcano plots showing the expression pattern of DEGs in control vs. treated panicle. IPC = IR 64, panicle, control; IPT = IR 64, panicle, treatment (drought); NPC = Nagina 22, panicle, control; NPT = Nagina 22, panicle, treatment.

Further analysis of the DEGs (>2-fold change, FDR ≤ 0.05) categorized into up- and downregulated genes in the four different comparison groups (Figure 7A), as well as representing them in volcano plots for significantly up- and downregulated genes in two comparison (IPC vs. IPT and NPC vs. NPT) groups, confirmed the role of upregulated genes in drought tolerance (Figure 7B).

Analyzing the top 50 DEGs significantly up- and downregulated in four different comparison groups clearly showed genotypic variations in the pattern of gene expression in response to reproductive-stage drought stress (Supplementary Figure S2). While many of the genes in NPC vs. NPT showed upregulated expression (compared to half upregulated and half downregulated in IPC vs. IPT), the genes downregulated in IR 64 on stress (IPC vs. IPT) were upregulated/more upregulated compared to that observed in IPC vs. IPT or IPT vs. NPT comparison group (Supplementary Figure S2). 

**Figure 8:**
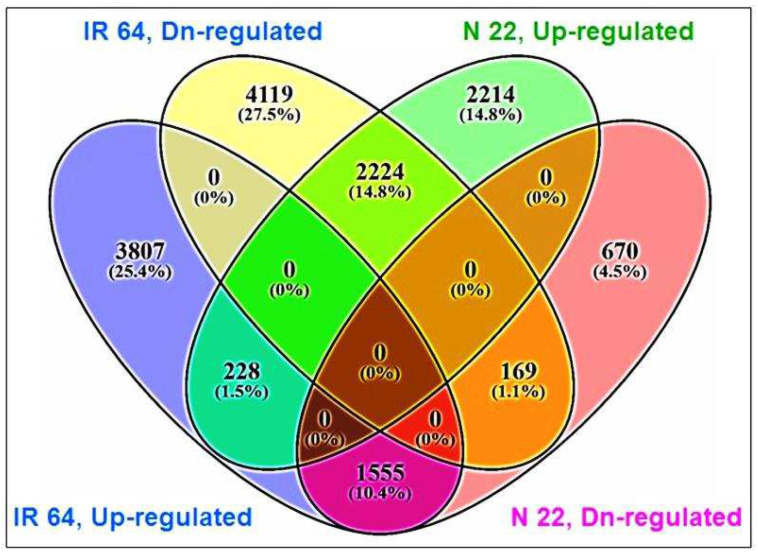
Four-way analysis of up- and downregulated differentially expressed genes (DEGs) in contrasting rice cultivars (N 22 and IR 64) in response to drought stress. DEGs were calculated for >2-fold change at FDR < 0.05.

Four-way analysis of the DEGs further confirmed that 2214 genes were upregulated exclusively in the panicle of N 22 under the stress, while 2224 genes upregulated in the panicle of N 22 were downregulated in the panicle of IR 64 (Figure 8, Supplementary Table S3). Only 228 genes were commonly upregulated in the panicle of both the rice cultivars under the stress. Moreover, 1555 genes downregulated in the panicle of N 22 under the stress were upregulated in the panicle of IR 64 under the stress. Only 169 genes were commonly downregulated in the panicle of both the rice cultivars at reproductive-stage drought stress (Figure 8). Many of the genes upregulated in the panicle of IR 64 were downregulated in N 22 (Supplementary Table S4). To our surprise, >14,500 genes were more upregulated, >15,000 genes were more downregulated, and >8000 genes showed no change in their expression in the panicle of N 22, compared to that in the panicle of IR 64 (IPT vs. NPT), in response to the reproductive-stage drought stress (Supplementary Table S5). Many of the genes were downregulated (>5-fold) in the panicle of N 22 under the stress including those for hypothetical proteins; however, some of the coding genes such as peptidase aspartic, fatty acyl-CoA synthetase, chitinase, etc. were also considerably (>9-fold) downregulated in the panicle of N 22 under the stress (Supplementary Table S6).

### 2.6. Function Enrichment Analysis of Differentially Expressed Genes

**Figure 9:**
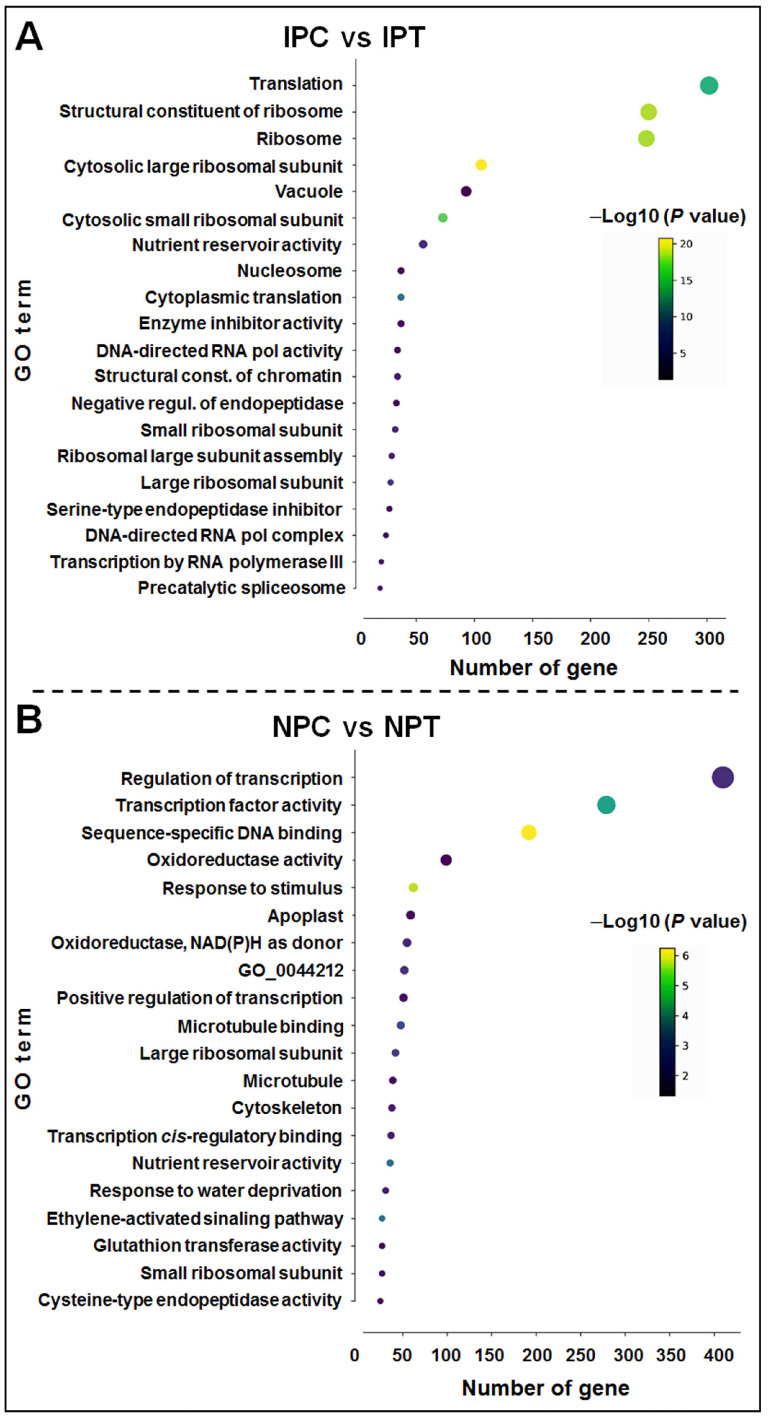
Gene ontology (GO) analysis of differentially expressed genes in the panicle of the contrasting rice cultivars in response to reproductive-stage drought stress: (**A**) IPC vs. IPT, (**B**) NPC vs. NPT. Only the top 20 GO terms, based on enrichment score [−Log10 (*p* value)] are presented. IPC = IR 64, panicle, control; IPT = IR 64, panicle, treatment (drought); NPC = Nagina 22, panicle, control; NPT = Nagina 22, panicle, treatment. The color of the dot depicts the enrichment score of the GO term, while the size of the dot represents the gene count for the term.

To gain insights into the role/function of DEGs between IPC vs. IPT and NPC vs. NPT (Supplementary Tables S3 and S4) functional enrichment analysis through gene ontology (GO) and KEGG pathway analyses were performed. In the panicle of N 22 at reproductive-stage drought stress (NPC vs. NPT), a total of 123 significant GO terms comprising 2444 genes, including those for transcriptional regulation (GO:0006355, 410 gene counts), DNA-binding transcription factor activity (GO:0003700, 279 gene counts), oxidoreductase activity (GO:0016709), positive regulation of transcription (GO:0045893), nutrient reservoir activity (GO:0045735), response to water deprivation (GO:0009414), water-channel activity (GO:0015250), and positive regulation of response to water deprivation (GO:1902584) (Supplementary Table S7). In contrast, the IPC vs. IPT comparison group showed a total of 119 GO terms comprising 2353 genes, including those for translation (GO:0006412, 302 gene counts), structural constituent of ribosome (GO:0003735), ribosome (GO:0005840), and cytosolic large ribosomal subunit (GO:0022625) (Supplementary Table S8). The top 20 enriched GO terms in IPC vs. IPT and NPC vs. NPT comparison groups are presented in Figure 9A,B. While the GO terms associated with regulatory functions (gene expression) were enriched in the case of N 22, the GO terms associated with the structural component (translation) were observed to be enriched in IR 64.

**Figure 10:**
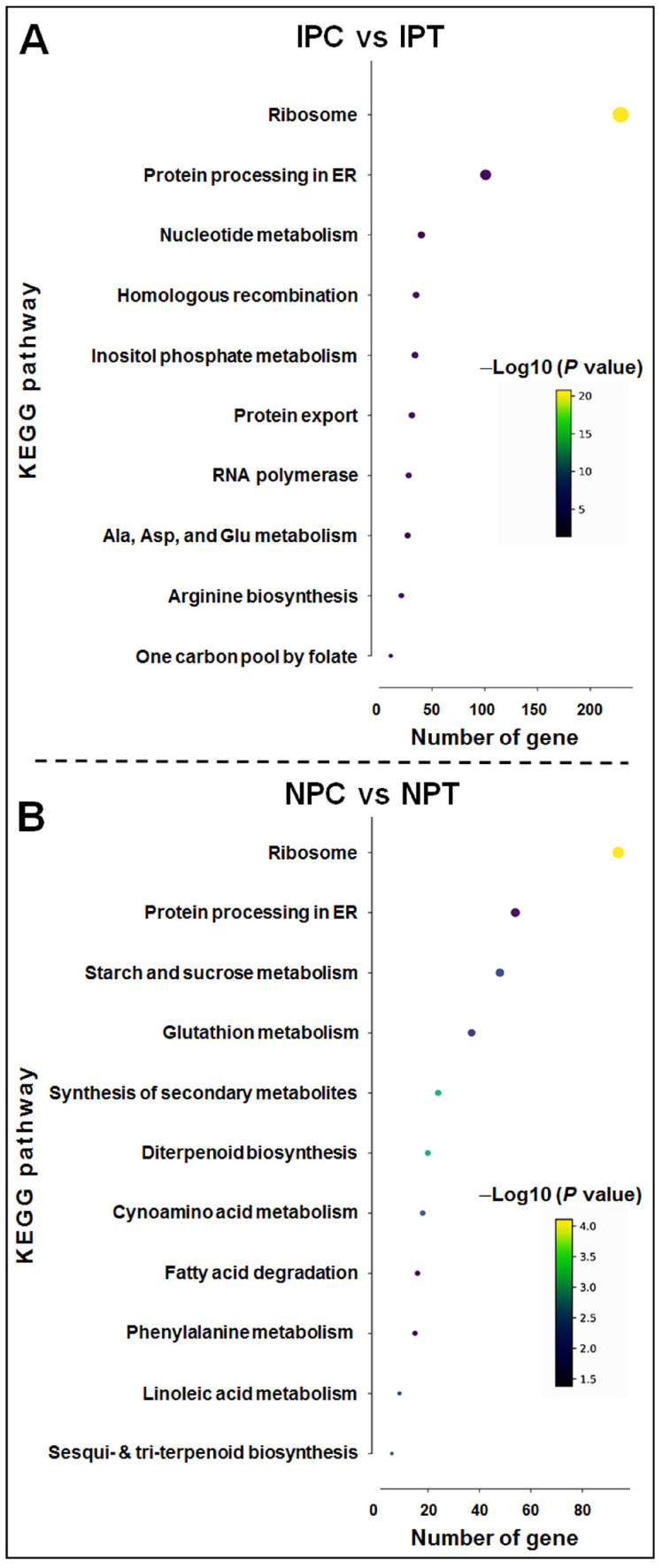
Kyoto Encyclopedia of Genes and Genomes (KEGG) pathway enrichment analysis in panicle of the contrasting rice cultivars in response to reproductive-stage drought stress. (**A**) IPC vs. IPT, (**B**) NPC vs. NPT. Only the top 20 enriched pathways based on enrichment score [−Log10 (*p* value)] are presented. IPC = IR 64, panicle, control; IPT = IR 64, panicle, treatment (drought); NPC = Nagina 22, panicle, control; NPT = Nagina 22, panicle, treatment. The color of the dot depicts the enrichment score of the pathway, while the size of the dot represents the gene count for the pathway.

Furthermore, to characterize the pathways involved in reproductive-stage drought tolerance in N 22, the DEGs were subjected to KEGG pathway enrichment analysis. In response to drought stress, significant enriched of the pathways including ribosome (map03010), protein processing in the endoplasmic reticulum (map04141), and nucleotide metabolism (map01232) was observed in the panicle of IR 64 under the stress (IPC vs. IPT) (Figure 10A). However, in the panicle of N 22 (NPC vs. NPT) the pathways including starch and sucrose metabolism (map00500), glutathione metabolism (map00480), synthesis of various secondary metabolites (map00999), phenylalanine metabolism (map00360) and linoleic acid metabolism (map00909) were enriched (Figure 10B). Thus, significant differences in enriched pathways between N 22 and IR 64 at reproductive-stage drought stress indicate differential metabolic pathway regulation in response to the stress.

### 2.7. Functional Annotation of Drought-Stress Responsive Genes Using MapMan

To categorize the DEGs involved in drought stress-related pathways, MapMan analysis was performed for the IPC vs. IPT and NPC vs. NPT comparison groups. Within the regulatory function terms, the DEGs associated with phytohormonal regulation, detoxification and redox signaling, secondary metabolites regulation, and transcription factor (TF)-mediated regulation of gene expression were more prominent.

#### 2.7.1. Phytohormone Biosynthesis and Signal Transduction

**Figure 11:**
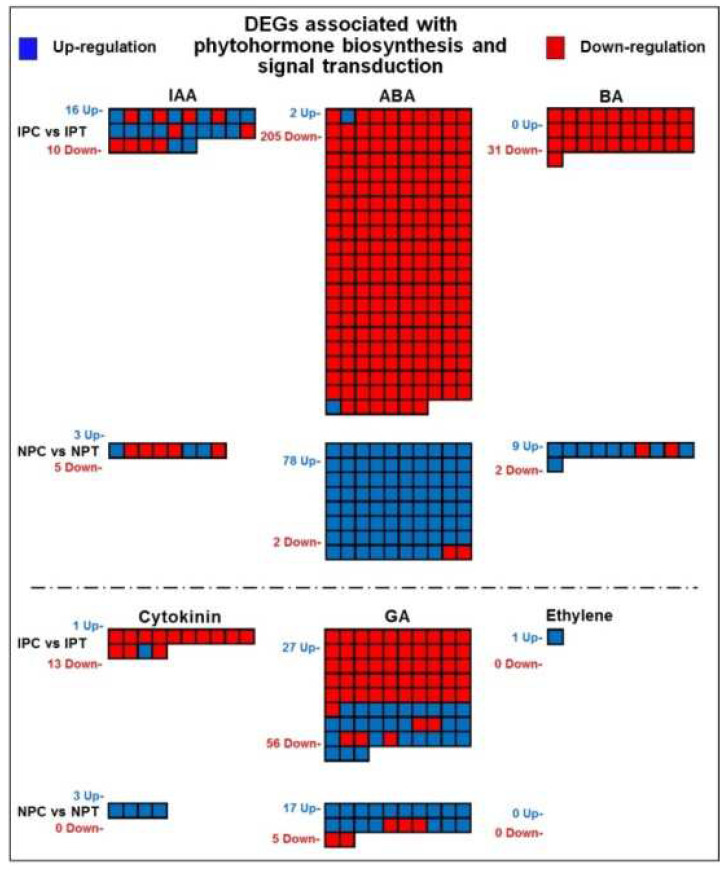
MapMan analysis of differentially expressed genes (DEGs) associated with phytohormone biosynthesis and signal transduction in a pairwise comparison (IPC vs. IPT and NPC vs. NPT) groups. The blue box represents upregulated and the red box represents the downregulated expression of the gene. IPC = IR 64, panicle, control; IPT = IR 64, panicle, treatment (drought); NPC = Nagina 22, panicle, control; NPT = Nagina 22, panicle, treatment; IAA = Indole acetic acid; ABA = Abscisic acid; BA = Brassinosteroids; GA = Gibberellic acid.

Comparative analysis of panicle for treatment over the control for N 22 (NPC vs. NPT) indicated 78 genes, involved in ABA biosynthesis, to be upregulated and 2 genes to be downregulated at reproductive-stage drought stress. In contrast, the DEGs for ABA biosynthesis in IR 64 (IPC vs. IPT) only genes were upregulated while 205 genes were downregulated in response to the drought stress. Moreover, 31 genes involved in brassinosteroid synthesis showed downregulated expression in IR 64 (IPC vs. IPT), while none of the genes was upregulated. In the case of N 22 (NPC vs. NPT), 9 genes involved in the brassinosteroid biosynthesis showed up-regulation while 2 genes were downregulated. Furthermore, the genes involved in biosynthesis and signal transduction of IAA, cytokinin, gibberellic acid (GA), and ethylene showed a contrasting pattern of regulation in the panicle of N 22 and IR 64 in response to the reproductive-stage drought stress (Figure 11).

#### 2.7.2. Redox Signaling and Detoxification of Reactive Oxygen Species

**Figure 12:**
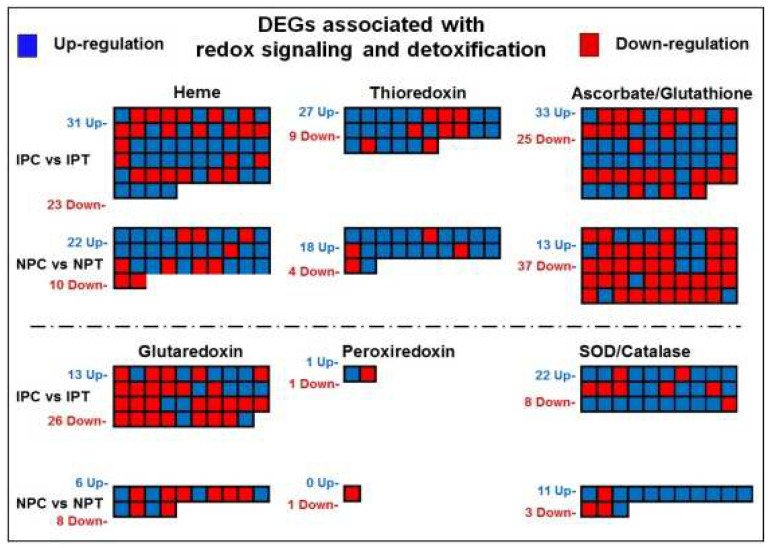
MapMan analysis of differentially expressed genes (DEGs) associated with redox signaling and detoxification pathways in a pairwise comparison (IPC vs. IPT and NPC vs. NPT) groups. The blue box represents upregulated and the red box represents the downregulated gene. IPC = IR 64, panicle, control; IPT = IR 64, panicle, treatment (drought); NPC = Nagina 22, panicle, control; NPT = Nagina 22, panicle, treatment and SOD = Superoxide dismutase.

The genes involved in redox signalling and scavenging of reactive oxygen species (ROS) by detoxification/scavenging reactions and maintaining redox homeostasis under drought stress in rice include thioredoxin (TRX), ascorbate (Asc), glutathione (GSH), glutaredoxin, dismutase, catalase (CAT), and heme, which are associated with enzymatic and non-enzymatic antioxidant machinery. Among the 54 DEGs associated with heme regulation in IR 64, 31 genes were up- and 23 were downregulated at reproductive-stage drought stress; whereas in N 22, up-regulation of 22 and down-regulation of 10 genes were observed (Figure 12). Moreover, upregulated expression of 13 genes and down-regulation of 37 genes in N 22 (compared to the up-regulation of 33 and down-regulation of 25 genes in IR 64) was observed for the DEGs associated with Ascorbate/Glutathione regulation. Furthermore, the DEGs associated with thioredoxin, glutaredoxin, and superoxide dismutase (SOD)/catalase also showed a contrasting pattern of regulation in the panicle of N 22 and IR 64 under the stress.

#### 2.7.3. Regulation of Secondary Metabolite Biosynthesis under Drought Stress

**Figure 13:**
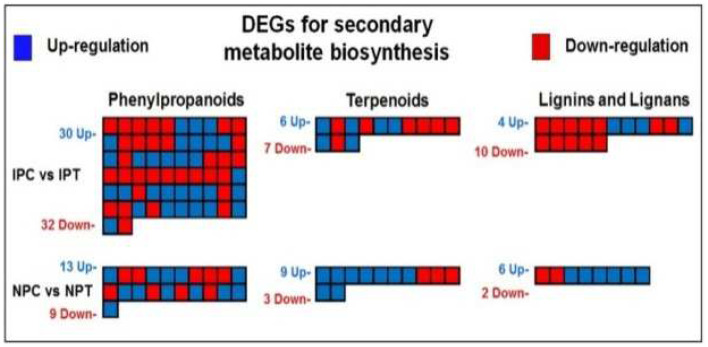
MapMan analysis differentially expressed genes (DEGs) associated with secondary metabolite biosynthesis in a pairwise comparison (IPC vs. IPT and NPC vs. NPT) groups. The blue box represents upregulated and the red box represents the downregulated gene. IPC = IR 64, panicle, control; IPT = IR 64, panicle, treatment (drought); NPC = Nagina 22, panicle, control; NPT = Nagina 22, panicle, treatment.

The DEGs associated with secondary metabolite biosynthesis regulation included 30 up- and 32 downregulated genes in IR 64, whereas 13 up- and 9 downregulated genes in N 22 for phenylpropanoids biosynthesis; 6 up- and 7 downregulated genes in IR 64, while 9 up- and 3 downregulated genes in N 22 for terpenoid biosynthesis; 4 up- and 10 downregulated genes in the panicle of IR 64, while 6 up- and 2 downregulated genes in the panicle of N 22 associated with lignins and lignans biosynthesis (Figure 13).

#### 2.7.4. Regulation through Differential Expression of Transcription Factors

A total of 793 differentially expressed transcription factors (TFs), belonging to 51 different TF families, were observed in two comparison (IPC vs. IPT and NPC vs. NPT) groups at reproductive-stage drought stress. Upregulated expression of the genes for TF families ERF, bHLH, NAC, WRKY, MYB, bZIP, C2H2, MYB-related, C3H, GRAS, and ARF was observed in the panicle of N 22 under the stress. Out of 335 TF genes upregulated (>1–27-fold) in N 22, only 16 were upregulated (4 comparatively more upregulated) in IR 64 (Supplementary Table S9). Moreover, 237 TF genes showed an up- and 359 TF genes showed downregulated expression in IR 64 under the stress. In N 22, only 92 TF genes showed downregulated expression in response to the reproductive-stage drought stress.

### 2.8. RT-qPCR Validation of the Differentially Expressed Genes

**Figure 14:**
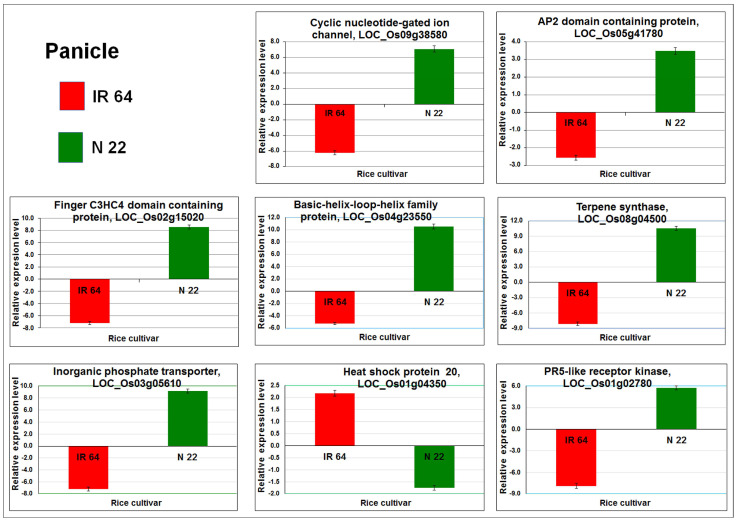
RT-qPCR validation of differential expression of the genes showing a contrasting pattern of expression in the panicle of the contrasting rice cultivars grown under reproductive-stage drought stress.

To verify the trustworthiness of the RNA-seq data, the expression level of 8 DEGs were validated by Reverse Transcription quantitative PCR (RT-qPCR). Comparative expression analysis of the selected DEGs from comparison (IPC vs. IPT and NPC vs. NPT) groups showed a similar expression pattern (Figure 14) as observed in the RNA-seq data analysis. Thus, the trustworthiness of the RNA-seq data was confirmed through RT-qPCR analysis.

## 3. Discussion

For the last decade, considerable advancements have been made in deciphering the regulatory mechanisms for drought tolerance in rice. Despite the recent advances in molecular and computational biology, a complex adaptive mechanism underlying drought stress tolerance at the agronomically most important growth stage (panicle initiation, grain filling), severely prone to drought stress, has yet been elusive. Rice, being a water-loving crop, possesses low water-use efficiency compared to other cereal crops [10,20]. Because of the changing climatic conditions and frequent occurrences of drought [9], it has become important to genetically improve rice to withstand drought stress at different developmental stages [22,24]. Identification of the master regulators for drought-responsive pathways using drought-tolerant and drought-sensitive rice cultivars/genotypes in response to the reproductive-stage drought stress might help in deciphering the genes, metabolic pathways, and regulatory networks responsible for drought-stress tolerance. Therefore, in the present study, a pair of well-known contrasting rice (IR 64, reproductive-stage drought-sensitive; N 22, drought-tolerant) cultivars [20] was used for comparative transcriptome analysis of panicle tissues at reproductive-stage drought stress.

### 3.1. Morpho-Physio-Biochemical Changes Affect Drought Tolerance

Imposition of drought stress caused reduced soil moisture content by 75% and relative water content of leaves reduced to 58–61% along with rolling-off/wilting of leaves (Figure 1), which ensured imposition of drought stress to the rice plants [20]. Reduction in total chlorophyll content in leaves of rice cultivars (Figure 2), and delayed panicle initiation due to the stress [20,25] confirmed responsiveness of the rice cultivars to reproductive-stage drought as well as suitability of the experimental materials for the study. Moreover, assessment of certain biochemical parameters (antioxidant activity, total phenolics content, and proline content) in different tissues (leaf, root, and panicle) from the contrasting rice cultivars at reproductive-stage drought stress (Figure 3) and agronomic performance of the rice cultivars ensured the right selection of experimental material for the study.

The agronomic performance of the rice cultivars was differentially affected in terms of a decrease in the number of panicles (comparatively more in IR 64) (Figure 4A), increased number of chaffy seeds (more in IR 64) (Figure 4B), and 1000-seed weight (more in IR 64) (Figure 4C), which ultimately resulted in a severe (>60%) reduction in the grain yield of IR 64, while only 33% reduction was recorded in case of N 22 (Figure 4D). This affirms the drought-tolerant nature of N 22 and right selection of the experimental materials/cultivars to decipher the genes/mechanisms/pathways involved in reproductive-stage drought tolerance in rice through genome-wide comparative transcriptome analysis.

### 3.2. Transcriptome Analysis Reveals the DEGs in Panicle under Drought Stress

Comparative transcriptome analysis of the libraries for contrasting rice cultivars grown under reproductive-stage drought stress revealed a distinct pattern of gene expression in IR 64 and N 22 (Supplementary Tables S3 and S4). MA plot analysis of all the expressed genes for comparison groups IPC vs. IPT and NPC vs. NPT indicated that certain additional genes were continuously expressed in N 22 (a drought-tolerant cultivar), but they were not expressed in IR 64 (a drought-sensitive cultivar) even under drought stress (Supplementary Figure S1A,B). Interestingly, many of the grey-spotted genes (showing no change) under stressed conditions over the control (NPC vs. NPT, Supplementary Figure S1B) got converted into green spots (upregulated genes in the panicle of N 22 over those expressed in the panicle of IR 64 under the stress) (Supplementary Figure S1D). More interestingly, some of the stress-associated genes were highly upregulated and only a smaller number of genes were downregulated even under control condition in panicle of N 22 (IPC vs. NPC, Supplementary Figure S1C) indicating that such genes are involved in quick responses (stress memory) of N 22 on the occurrence of drought stress. Thus, the increased number/upregulated expression of genes is mainly responsible for making N 22 a drought-tolerant rice cultivar (Figure 8).

### 3.3. Functional Categorization Revealed the GO terms and Metabolic Pathways

The GO terms enriched in panicles of N 22 at reproductive-stage drought included ‘regulation of transcription’, ‘transcription factor activity’, ‘response to stimulus’, ‘nutrient reservoir activity’, and ‘response to water deprivation’ (mostly the regulatory terms), whereas in the case of IR 64 the most significantly enriched GO terms included ‘translation’, ‘ribosome’, ‘vacuole’, etc. (mostly structural/functional components) in response to the stress (Figure 9 and Figure 10). Unlike in N 22, the GO term associated with ‘response to water deprivation’ or ‘water channel activity’ was not observed to be enriched in IR 64 in response to the stress, indicating the downregulated expression of drought-associated genes in IR 64 during the stress. Moreover, significant enrichment of drought-responsive GO terms such as oxidoreductase activity, response to stimulus, glutathione transferase activity, phenylpropanoid metabolic process, etc. (defensive responses) in N 22 under the stress must have complementary effects for drought tolerance [11,17,22,26]. KEGG pathway enrichment analysis for the DEGs in the panicle of contrasting rice cultivars under the drought stress indicated enrichment of drought-responsive metabolic (protein processing, sucrose and starch metabolism, glutathione metabolism, secondary metabolites synthesis, and phenylalanine and linoleic acid metabolism) pathways in N 22, while enrichment of only a few pathways (nucleotide metabolism and amino acid metabolism) in IR 64 under the stress, suggesting their role in making N 22 a better performer under the stress (Figure 10).

### 3.4. Phytohormone Signaling Plays Important Role in Drought Tolerance

Phytohormone biosynthesis and signaling play important role in plant defense against abiotic stress. Our findings on the upregulated expression of several genes associated with the biosynthesis of phytohormones (ABA, BA, auxin, and GA) in response to the stress in N 22 (Figure 11) are in agreement with those reported earlier [27,28,29,30]. ABA has been well-known to be an important phytohormone to regulate plant adaptive responses to abiotic stress that enhances ROS production and triggers enzymatic as well as non-enzymatic antioxidant defense systems [1,11,18,24,31,32]. Moreover, several genes involved in ABA synthesis/regulation have been reported to be upregulated in plants under drought stress [18,33]. Our findings on a higher number of upregulated genes related to ABA synthesis in panicle of N 22 in response to drought stress under the stress (Figure 11) corroborates with the earlier reports. Furthermore, earlier studies suggest that under drought stress brassinosteroids play important role in detoxifying the oxidative damage by up-regulating the expression of the genes involved in ROS scavenging and redox signalling in association with other phytohormones such as ABA [27,28,29,34]. Our results on enhanced expression of genes involved in biosynthesis/signaling of brassinosteroids in the panicle of N 22, and upregulated expression of none of the genes in IR 64, under the stress (Figure 11), corroborate the earlier findings. This can be considered as one of the important strategies adopted by N 22 in mitigating the adverse effects of reproductive-stage drought stress on panicle.

### 3.5. Redox Homeostasis/ROS Scavenging Contribute to Drought Tolerance

Enzymatic and non-enzymatic antioxidant defense systems play important roles in combating the deleterious effects of drought stress. Our findings on significant increase in the expression of genes coding for enzymes such as catalase and superoxide dismutase in the rice cultivars under the stress (Figure 12) is in agreement with earlier reports on scavenging of ROS [12,26,35]. Moreover, our observation on upregulated expression of the genes involved in phenylpropanoid biosynthesis in the rice cultivars under the stress corroborates with the earlier reports [22,36]. Cell wall remodeling and modulation of osmotic potential within the cell are some other strategies adopted by plants for subsistence under environmental stresses [37,38,39]. We observed upregulated expression of the genes involved in regulating lignins and lignans metabolism in the rice cultivars, particularly in N 22, under the stress (Figure 13), which is in agreement with the earlier report [40]. This is another important strategy adopted by N 22 to complement mitigating the adverse effects of reproductive-stage drought stress on seed development/yield. Thus, the genes associated with redox homeostasis, ROS detoxification, antioxidant defense, and various secondary metabolite biosynthesis pathways complement stress-adaptive modulations under the stress.

### 3.6. Transcription Factors Modulate Expression of Stress-Associated Genes

Considerably upregulated expression of genes for several transcription factors (TFs) including ERF, bHLH, NAC, WRKY, MYB, bZIP, C2H2, MYB-related, C3H, GRAS, ARF, etc. in the panicle of N 22 at the reproductive-stage drought (Supplementary Table S9) corroborates with the earlier reports [11,24,41,42,43,44,45,46]. Significant up-regulation of TFs affecting drought-responsive genes (involved in cellular response to stimulus, secondary metabolite biosynthesis, glutathione metabolism, auxin and ethylene biosynthesis, and response to dehydration/water deprivation) in the panicle of N 22 suggests their important roles in imparting drought stress tolerance.

### 3.7. Proposed Model for Drought Tolerance in Rice

**Figure 15:**
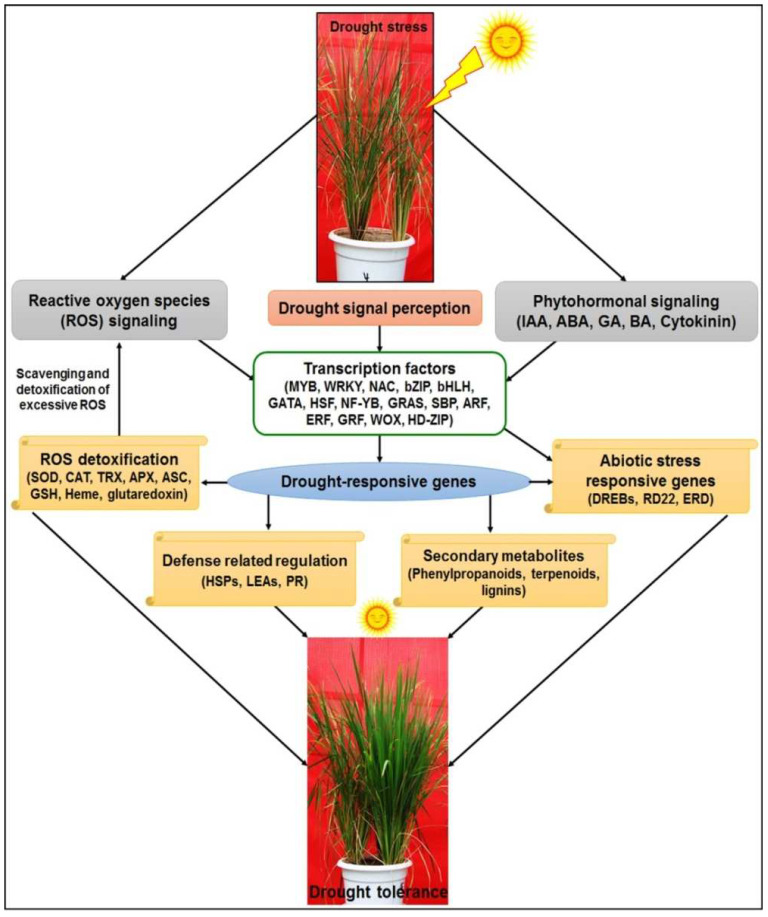
Proposed model for drought-induced pathways activated in rice for protection against reproductive-stage drought stress.

Based on our findings on the expression of genes associated with reproductive-stage drought tolerance in rice, particularly those for TFs and regulatory pathways, we propose a pictorial model showing the essential processes associated with drought stress signal perception, phytohormone and redox signaling, activation of TFs, and drought stress-associated defensive genes/processes (Figure 15). Functional validation of the drought-responsive genes and deciphering the molecular/regulatory pathways responsible for the reproductive-stage drought stress tolerance would provide valuable insights necessary for genetic manipulation of rice towards the development of climate-smart rice, particularly for direct-sown conditions [20], needed for the sustainable production of rice under changing climatic conditions with zero/negative emission agriculture for better ecological integrity.

## 4. Materials and Methods

### 4.1. Plant Materials and Drought Stress Treatment

Mature seeds of two contrasting rice cultivars [Nagina 22, drought tolerant; IR 64, sensitive to reproductive stage (reproductive stage) drought] were grown in pots in a net-house under natural conditions during the *Kharif* season (July–October) at the experimental farm of ICAR-Indian Agricultural Research Institute, New Delhi, India. Seedlings were raised in a nursery, followed by uprooting 25-day-old seedlings and transplanting them into pots (12″ diameter) filled with puddled soil. For raising the nursery, mature seeds were directly sown in pots filled with soil (soil moisture content ~24% *w*/*w*). One set of plants (consisting of 9 pots each with 3 rice plants) was grown as a control (irrigated on an alternate day with tap water). In contrast, another set was imposed with drought stress just before the initiation of flowering (at the reproductive stage). Drought stress was imposed by withholding irrigation for 4–5 days before panicle initiation (65 days after transplanting of N 22 cultivar). The level of stress was assessed by measuring soil moisture content (reduced to ~6%) and relative water content (dropped down to ~58%) of leaves. Then, immature panicles (still wrapped inside the flag leaf) were collected from the rice cultivars grown under control and drought conditions (Figure 1), snap-frozen in liquid nitrogen, and stored at −80 °C for further downstream processing.

### 4.2. Estimation of Biochemical Parameters

For estimations of various biochemical parameters, different tissues (0.5 g) freshly collected from the rice plants grown under control as well as drought stress were ground into a fine powder with the help of liquid nitrogen using a mortar and pestle to prepare the sample extract. The antioxidant potential of the rice extracts was measured using the DPPH scavenging method following the procedure described by [47]. Reduction in DPPH radical was determined at 515 nm until 1 h when a stable value was obtained. To determine total phenolics content (TPC) in the tissue samples, the procedure described by [48] was followed with slight modifications. Briefly, 1.0 g of the fresh tissue was extracted in 5 mL of 80% methanol. To 1.0 mL of the extract, 0.5 mL of 10% Folin–Ciocalteu reagent (*v*/*v*), 7.5 mL ddH_2_O followed by 1.5 mL of 20% sodium carbonate was added. Then, absorbance was recorded at 755 nm using a spectrophotometer. Gallic acid was used to prepare a phenol standard curve and TPC was expressed as mg of gallic acid equivalents per gram of the tissue. Proline content in the tissue samples was estimated by the sulfosalicylic acid method [49]. The total chlorophyll content in the leaf (0.5 g) was estimated using the dimethyl sulfoxide (DMSO) procedure described by Hiscox and Israelstam [50]. Leaves were cut into small (5 mm) pieces, submerged in 2 mL DMSO, and incubated for 20 min at 60 °C for chlorophyll extraction. The tissues were added with another 2 mL of DMSO and incubated again for 20 min at 60 °C. Finally, the extracts were pooled together and absorbance of the extract was recorded at 645 and 663 nm against DMSO as blank using a spectrophotometer. Total chlorophyll content on a dry weight (DW) basis was calculated using the formula:

Total chlorophyll content (mg/g DW) = DMI (20.2 × A645) + (8.02 × A663), Where DMI = Dry matter index (Dry weight/fresh weight) of the plant tissue.

### 4.3. RNA Extraction, cDNA Library Preparation, and Transcriptome Sequencing

Total RNA was extracted from the tissue (panicle) samples collected from the plants of both the rice cultivars grown under control and drought stress conditions using TRIzol reagent (Invitrogen, Carlsbad, CA, USA) following the protocol described by the manufacturer. Subsequently, RNA was treated with DNase I (QIAGEN, Hilden, Germany) to eliminate any contaminating DNA. Integrity/degradation of RNAs was checked on 1.2% agarose gel and then the quality of RNA samples was determined using Qubit 4. Only a high-quality total RNA (1 µg) sample (OD^260^/_280_ = ~2.0, OD^260^/_230_ ≥ 2.0, RIN ≥ 6.0, 28S:18S ≥ 1.0) was used as input material to construct the RNA-seq library. In total, 12 libraries were prepared for the panicles collected from both the rice (IR 64 and N22) cultivars grown under control and drought conditions in three replications following the procedure described earlier by Kumar et al. (2021). The libraries were named IPC (panicle from control plants of IR 64), IPT (panicle from drought-treated plants of IR 64), NPC (panicle from control plants of N 22), and NPT (panicle from drought-treated plants of N 22). The libraries were got sequenced at Illumina HiSeq 2500 platform using PE-150 bp chemistry by Macrogen (Seoul, Republic of Korea). Raw sequence data were submitted to the NCBI under the BioProject Submission ID SUB12273896 and used for bioinformatic analyses. 

### 4.4. Raw Data Processing and Mapping

Read quality check was performed using FastQC followed by trimming of raw reads using Trim Galore to remove the adaptor, poly-N, and low-quality reads, followed by an after-trim quality check with FastQC. The raw sequence data were processed using pySeqRNA (a python package for transcriptome data analysis, http://bioinfo.usu.edu/pyseqrna/, accessed on 23 December 2022) with default parameters [51]. Feature counts were performed using featureCounts [52]. The raw read counts were normalized for reads per kilobase of the exon model per million mapped reads using the *Normalization* module of pySeqRNA. Clean reads were mapped on *Oryza sativa* Japonica reference IRGSP-1.0 genome (https://plants.ensembl.org/Oryza_sativa/Info/Index, accessed on 28 October 2022) using STAR aligner [53].

### 4.5. Identification of Differentially Expressed Genes

DESeq2 [54] was used to identify the differentially expressed genes (DEGs) between different cultivars or conditions, which provides digital gene expression data based on statistical analysis for the negative binomial distribution model. To account for the false discovery rate, the *p* value was adjusted using the Benjamini and Hochberg method [55]. Genes with ≥2-fold change (|log2Ratio| > 1) having statistically significant abundance difference (FDR < 0.05) were considered as DEGs.

### 4.6. Functional Enrichment Analysis

Functional enrichment of the DEGs was performed using *the Gene_ontology* module of pySeqRNA to find enriched GO terms and the *Pathway* module was used to find enriched KEGG pathways in the DEGs. The pySeqRNA uses BioMart in the backend for Gene Ontology and KEGG pathway enrichment analyses. The top 20 enriched terms, based on enrichment score [−log_10_ (*p*-value)], were plotted in a bar plot.

### 4.7. Functional Annotation of DEGs Using MapMan

To further characterize the DEGs, MapMan visualization was used which identifies gene families that may play essential roles and visualizes data on diagrams. All the CDS sequences were classified into functional hierarchical classifications (BINs) using Mercator [56].

### 4.8. Validation of Differential Gene Expression by RT-qPCR

To validate the differential expression pattern of genes (as visualized on transcriptome analysis) in different cultivars/conditions, the expression of some of the randomly selected genes (playing important roles in the adaptation of rice to drought stress) was validated by RT-qPCR. The validation was performed on an independent set of samples (from the plants grown under a similar experimental plan in a subsequent year) to confirm their altered expression in the contrasting cultivars under different conditions. The total RNAs isolated from panicle of N 22 and IR 64 grown under control and drought conditions were subjected to DNase I treatment, followed by reverse transcription using Superscript II (Invitrogen). RT-qPCR validation of expression pattern for the selected genes was performed in three biological and three technical triplications using SYBR Green PCR Master Mix kit (Applied Biosystems, Foster city, CA, USA) following the manufacturer’s instructions using QIAquant 96 5plex machine (Qiagen, Hilden, Germany). Details of the primers used for RT-qPCR are listed in Supplementary Table S10. The PCR amplification was performed in 10 µL reaction volume and the thermal cycler was programmed for initial denaturation at 95 °C for 3 min, followed by 40 cycles each of denaturation at 94 °C for 10 s, annealing at 60 °C for 15 s, and extension at 72 °C for 20 s. Data collection was set at the end of every extension step and the data was used for melt curve analysis. The relative gene expression was determined using the 2^−ΔΔCT^ method. Actin and tubulin genes were used as internal reference genes. The relative expression level represents fold change in the expression of the target gene and the error bar represents the standard deviation (±SD).

### 4.9. Statistical Analysis

Most of the physio-biochemical experiments were carried out with three biological/technical replications. Statistical analysis was performed by analysis of variance (ANOVA), Post hoc Tukey test or Duncan’s multiple range test (DMRT) at *p* ≤ 0.05 were used to compare the means of treatments. In the case of RT-qPCR analysis, the relative expression represents the fold change in the expression of the gene. The mean values followed by different lower-case letters are significantly different (*p* ≤ 0.05). The error bars represent the standard deviation (±SD). Mapping of the cleaned RNA-seq data was performed after normalization to the *r*eads *p*er *k*ilobase of transcript per million (RPKM) value. Differential expression of genes (DEGs) was determined for >2-fold change in expression with the false discovery rate (FDR) ≤ 0.05.

## 5. Conclusions

Drought tolerance is a multigenic complex trait that requires interplay of a variety of pathways, transcription factors, and genes. Some of the rice landraces/cultivars have evolved adaptive strategies to protect themselves from drought stress; however, the high-yielding rice varieties, being more input-responsive, lack such ability. Therefore, we conducted comparative physiological, biochemical, and molecular analyses of contrasting rice cultivars to decipher the candidate genes, transcription factors, mechanisms, and pathways responsible for drought tolerance. The present study suggests that stress signalling, redox homeostasis, antioxidant activity, biosynthesis of secondary metabolites, linoleic acid metabolism, ABA biosynthesis, and transcription factors are mainly responsible for the observed reproductive-stage drought stress tolerance in N 22. Upregulated expression of some of the stress-responsive genes in panicle of N 22, even under control (no drought) conditions, must be responsible for quick and effective protection of this cultivar from the drought stress. The information might be useful in genetic improvement of rice through genetic and/or molecular approaches towards the development of drought-tolerant rice cultivars which can endure intermittent drought of varying intensity due to the changing climatic conditions. However, functional validation of the drought-responsive candidate gene(s) would be necessary before utilizing their potential in rice genetic improvement programs.

## Figures and Tables

**Table 1:** Summary of data alignment statistics for control and drought samples from contrasting rice cultivars.

Sample Name	Raw Reads	Clean Reads	Aligned Reads	Mapping Efficiency (%)
IPC_1	48,966,509	48,836,418	46,192,763	94.59
IPC_2	53,966,608	53,824,700	50,887,777	94.54
IPC_3	58,966,608	58,712,210	53,254,093	94.47
IPT_1	51,707,279	49,000,200	41,855,876	85.42
IPT_2	52,207,282	49,475,010	42,261,648	85.42
IPT_3	52,207,282	49,475,010	42,261,648	85.42
NPC_1	54,547,228	53,298,510	49,754,853	93.35
NPC_2	45,738,722	42,088,332	39,179,553	93.08
NPC_3	45,312,718	44,272,050	41,333,550	93.36
NPT_1	55,322,536	55,124,818	51,637,008	93.67
NPT_2	65,243,028	64,132,208	59,876,829	93.36
NPT_3	60,373,548	60,267,168	56,349,061	93.50

(IPC = IR 64, panicle, control; IPT = IR 64, panicle, drought-treated; NPC = N 22, panicle, control; NPT = N 22, panicle, drought-treated).

## Data Availability

RNA-Seq raw data are available at NCBI Sequence Read Archive (SRA) database (https://www.ncbi.nlm.nih.gov/sra, accessed on 19 December 2022) under the BioProject Submission ID SUB12273896.

## Supplementary Materials

The following supporting information can be downloaded at: https://www.mdpi.com/article/10.3390/ijms24021002/s1.
