# Supplementary material for: Transcriptome and Physio-Biochemical Profiling Reveals Differential Responses of Rice Cultivars at Reproductive-Stage Drought Stress
Source: Int J Mol Sci. 2023 Jan 5;24(2):1002. doi: 10.3390/ijms24021002 (PMC9863700; doi:10.3390/ijms24021002)

Supplementary Figure S1: MA plot depicting up- and down-regulated as well as not significantly changed expression of genes under control and drought stress in panicle of contrasting rice (N 22 and IR 64) cultivars in four different comparison groups: (**A**) IPC vs IPT, (**B**) NPC vs NPT, (**C**) IPC vs NPC, (**D**) IPT vs NPT.

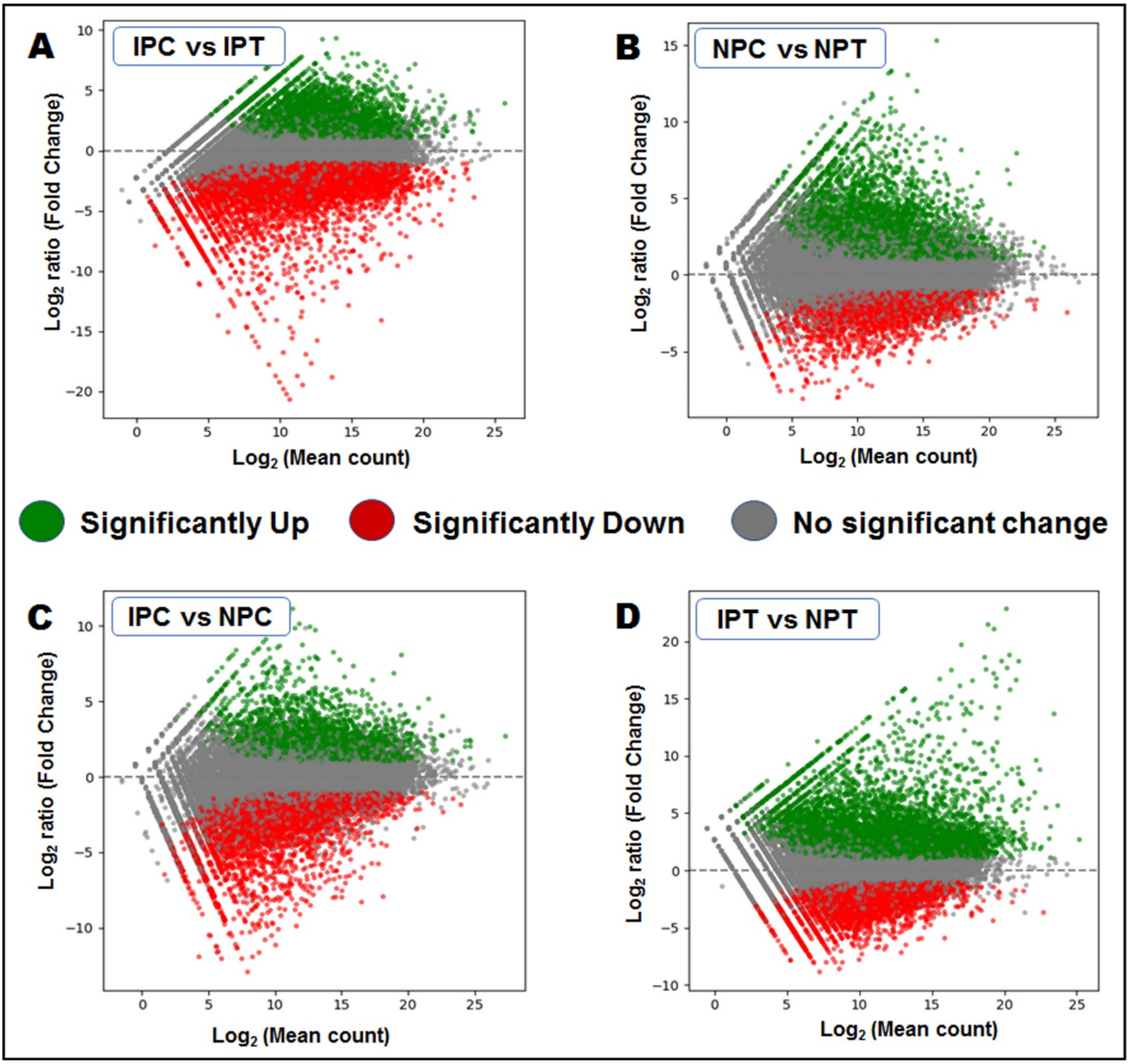

Supplementary Figure S2: Heat map showing the top 50 differentially (>2-fold change, FDR ≤0.05) expressed genes under control and drought stress in panicle of contrasting rice cultivars, presented in four pairwise comparisons. IPC= IR 64, panicle, control; IPT= IR 64, panicle, treated (drought); NPC= Nagina 22, panicle, control; NPT= N 22, panicle, treated.

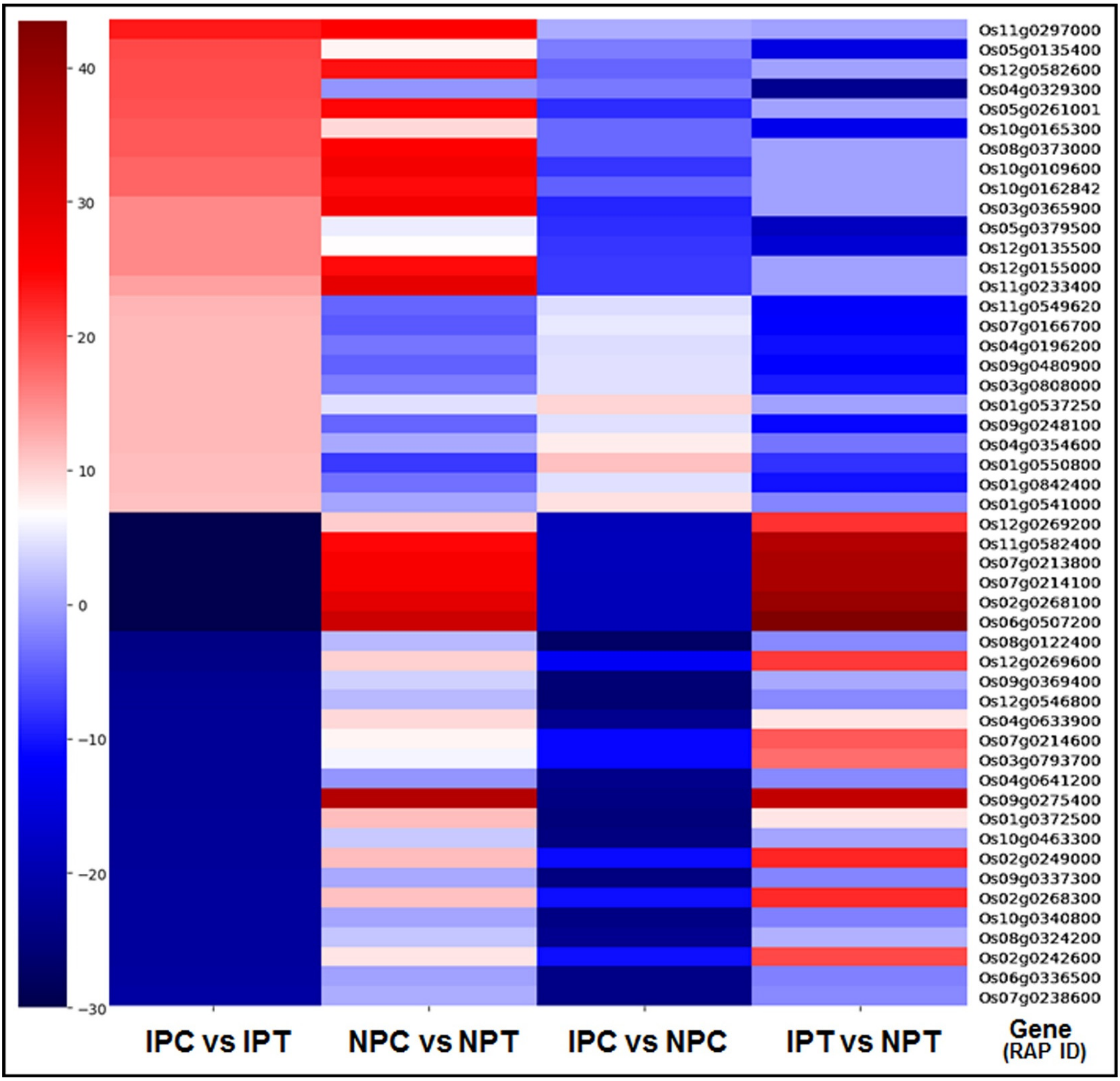

Supplement: Supplementary file 1 [file ijms-24-01002-s001.zip › Supplementary Figure (Revised) IJMS.pdf]
