# Supplementary material for: Transcriptome and Physio-Biochemical Profiling Reveals Differential Responses of Rice Cultivars at Reproductive-Stage Drought Stress
Source: Int J Mol Sci. 2023 Jan 5;24(2):1002. doi: 10.3390/ijms24021002 (PMC9863700; doi:10.3390/ijms24021002)
Supplement: Supplementary file 1 [file ijms-24-01002-s001.zip › Supplementary Table S10.pdf]

Supplementary Table S10. List of primers used for RT-qPCR validation of randomly selected differentially expressed genes in the panicle of contrasting rice (IR 64, reproductive stage drought sensitive; N 22, drought tolerant) cultivars.

**Gene Name (RAP ID)**	**Gene ID**	**Forward Primer (5′→ 3′)**	**Reverse Primer (5′→ 3′)**
Cyclic nucleotide-gated ion channel (Os09g0558850)	LOC_Os09g38580	GGACATTTGCGGACTTGTTT	ACCTGAGGAAGAGGGATCGT
AP2 domain containing protein (Os05g0497300)	LOC_Os05g41780	CCCGTATCGCACCCTTACTA	GTGATGGTGCCAGATCCAC
Finger (C3HC4) type domain containing protein (Os02g0248200)	LOC_Os02g15020	GAAGCAAGTCGGGGAGGT	CTGTGGCTTTCACCACGAT
Basic-helix-loop-helix family protein (Os04g0301500)	LOC_Os04g23550	GACGCAGGATGACTCCAACT	CGCTCCATGAGGATGTTCTT
Terpene synthase (Os08g0139700)	LOC_Os08g04500	GCACCTTGGAAGAAAGCAAG	AGCATCTCGTAGCGTCGATT
Inorganic phosphate transporter (Os03g0150800)	LOC_Os03g05610	AGGTGGAGGAGGAGAAGCTC	CTGGAACAGGTTCTGGCTGT
Heat shock protein (HSP20) (Os01g0135900)	LOC_Os01g04350	TGTTTGATACGTTGGCGTTC	GCACCTCCTCCTTCTTCACC
PR5-like receptor kinase (Os01g0117200)	LOC_Os01g02780	CAGAATCCACCACATCAACG	ACCCTTGGTGCAGGTAGTTG
Actin gene	LOC_Os03g50885	TTGCTGACAGGATGAGCAAG	TGGAATGTGCTGAGAGATGC
β-tubulin gene	LOC_Os01g59150	GCTGACCACACCTAGCTTTGG	AGGGAACCTTAGGCAGCATGT
